# TLC-smartphone in antibiotics determination and low-quality pharmaceuticals detection†

**DOI:** 10.1039/d1ra01346g

**Published:** 2021-05-26

**Authors:** Asmaa G. Gad, Yasmin Mohammed Fayez, Khadiga M. Kelani, Amr M. Mahmoud

**Affiliations:** Analytical Chemistry Department, Faculty of Pharmacy, Modern University for Technology and Information Cairo Egypt; Analytical Chemistry Department, Faculty of Pharmacy, Cairo University El-Kasr El-Aini Street 11562 Cairo Egypt amr.bekhet@pharma.cu.edu.eg

## Abstract

Thin layer chromatography (TLC) is a powerful and simple technique for screening and quantifying low quality and counterfeit pharmaceutical products. The detection methods used to detect and quantify separate analytes in TLC ranges from the densitometric method to mass spectrometric or Raman spectroscopic methods. This work describes the development and optimization of a simple and sensitive TLC method utilizing a smartphone CCD camera for verification of both identity and quantity of antibiotics in dosage form, namely ofloxacin and ornidazole. Mixtures of ofloxacin and ornidazole were chromatographed on a silica gel 60 F_254_ plate as a stationary phase. The optimized mobile phase is *n*-butanol : methanol : ammonia (8 : 1 : 1.5 by volume). Iodine vapor has been used as a “universal stain” to visualize the spots on the TLC plates in order to obtain a visual image using the smartphone camera and a desk lamp as an illumination source, thus eliminating the need for a UV illumination source. The recorded images were processed to calculate the *R*_f_ values (*R*_f_ values for ofloxacin and ornidazole were 0.12 and 0.76, respectively) which provide identity of the drugs while spot intensity was calculated using a commercially available smartphone app and employed for quantitative analysis of the antibiotics and “acetaminophen” as an example of a counterfeit substance. The smartphone TLC method yielded a linearity of ofloxacin and ornidazole in the range of 12.5–62.5 μg/band and 500–1000 μg/band, respectively. The limit of detection was found to be 1.6 μg/spot for ofloxacin and 97.8 μg/spot for ornidazole. The proposed method was compared with the bench top densitometric method for verification using a Camag TLC Scanner 3, the spot areas were scanned at 320 nm. The *R*_f_ value of ofloxacin and ornidazole was calculated to be 0.12 and 0.76, respectively. The densitometric method yielded a linearity of ofloxacin and ornidazole in the range of 5–40 μg/band and 5–50 μg/band, respectively. The limit of detection was found to be 0.8 μg/spot for ofloxacin and 1.1 μg/spot for ornidazole. The proposed method has been successfully applied for the determination of ofloxacin and ornidazole present in more than one pharmaceutical dosage form and was comparable to the densitometric method.

## Abstract 2

Low-quality and counterfeit pharmaceutical detection has been performed based on the processing of an iodine stained TLC plate image captured by a smartphone CCD camera.

## Graphical abstract

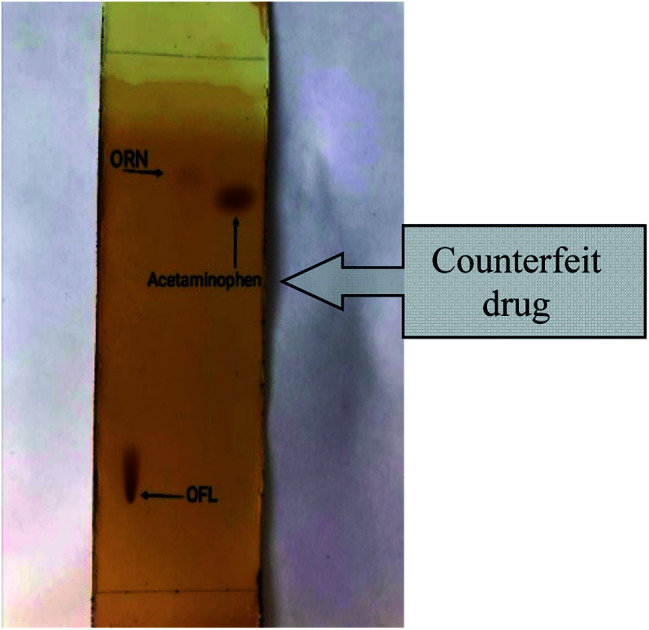

## 1. Introduction

Low-quality medicine is a major problem for patients in developing countries, and it is estimated that 1 in 10 medical products in low- and middle-income countries is substandard or falsified.^1–3^ Counterfeit medications may contain falsified medication where no active pharmaceutical ingredient (API) is added or substandard APIs (wrong amount of the correct active ingredient), substituted by other cheaper and incorrect APIs (for example acetaminophen)^4^ or even toxic substances.^5^ Low-quality pharmaceuticals have a negative impact on the patient's health as the incorrect drug might make the patient's condition worse, or even kill them. Moreover, for antibiotic dosage form, the absence or low active ingredient content may result in the emergence of drug-resistant bacterial strains.^6^ Antimicrobial resistance (AMR) is the ability of a microbe to resist the effects of medication that once could successfully treat the microbe.^7^ The term antibiotic resistance is a subset of AMR, as it applies only to bacteria becoming resistant to antibiotics.^8,9^ Resistant microbes are more difficult to treat, requiring alternative medication, and combination or higher doses of antimicrobials. These approaches may be more expensive, more toxic or both.^10^

For the developing countries there is an urge for developing rapid, robust and low-cost techniques for identification of low-quality pharmaceuticals. Ideally, such detection technique can be able to detect falsified or substandard medicines. Thin layer chromatographic (TLC) method is ideal for this task and has been employed for detecting counterfeit medication, recent advances have been recently reviewed.^11^ TLC (and HPTLC) can be regarded as a simple but, in many cases, effective technique with similar performance to HPLC. TLC detection methods enable both qualitative information by utilizing the characteristic *R*_f_ values; and quantitative data by various techniques.^12^ Recently, cheap and widely available detection methods such as scanner, smartphones CCD camera,^13^*etc.* have been employed. The detectors capture the color produced on the TLC plate and gauge the intensity of color using image-processing software. The intensity is then computed in regression equation obtained from calibration curve to get the concentration of the drug.^14^ CCD smartphone cameras in particular are gaining more interest as detectors for colorimetric and TLC plates. Smartphone has been used for reading TLC plates to detect counterfeit drugs and even detection of illicit substance (*i.e.* cocaine) under UV lamp as source of illumination to visualize the analyte of interest.^15,16^ Using UV lamp as a visualization method for TLC adds extra cost for detection in developing and resource-limited areas; moreover, it is only limited to aromatic and conjugated compounds. Iodine has been used as universal method for TLC plate visualization, with the advantages of being widely available, cheap and “semi-destructive” as complexation is reversible and I_2_ will eventually evaporate. Moreover, there are indicators for visualizing the TLC plate that are specific for certain functional groups, such as nitro group like (acidified potassium permanganate, methanolic potassium hydroxide). It's worth mentioning that an innovative approach has been recently developed to visualize HPTLC plates using multiple illumination sources (in both UV and visible range) and images collected by smartphone CCD camera followed by data manipulation utilizing chemometrics, thus enabled HPTLC clustering/fingerprint.^17^

**Fig. 1:**
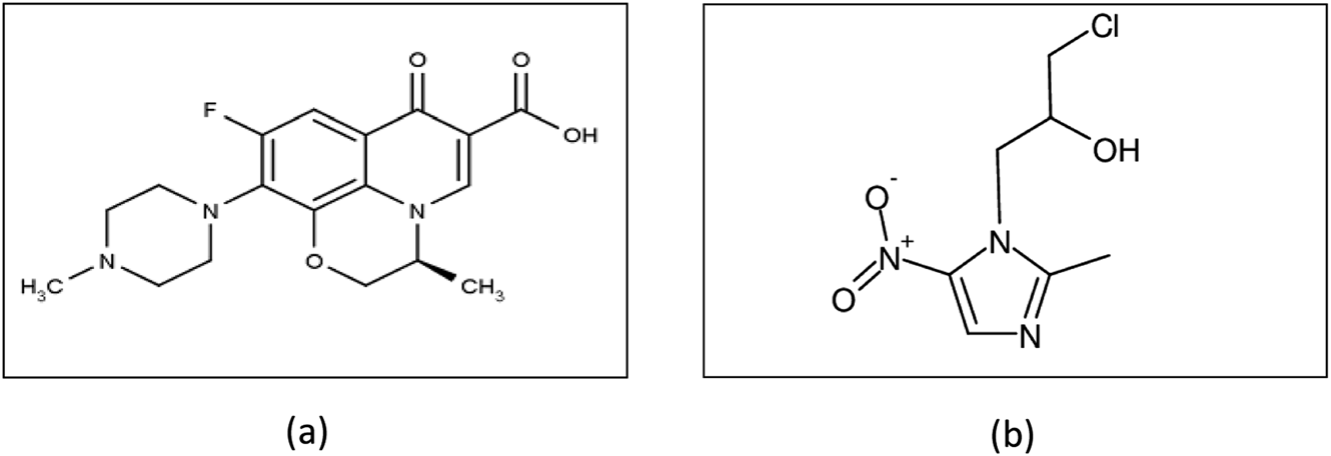
(a) Chemical structure of ofloxacin and (b) chemical structure of ornidazole.

Ofloxacin is a broad spectrum antibiotic belonging to the fluoroquinolone class,^18,19^ its structure is shown in Fig. 1a. It inhibits bacterial cell division by inhibiting DNA gyrase, a type II topoisomerase, and topoisomerase IV, which is an enzyme necessary to separate replicated DNA.^20^ Ornidazole is an antibiotic of the imidazole class;^21^ its structure is shown in Fig. 1b. Ornidazole enters the cell by diffusion where the nitro group is reduced by redox proteins present only in anaerobic organisms to reactive nitro radical which exerts cytotoxic action by damaging DNA and other critical biomolecules.^22,23^ DNA helix destabilization and strand breakage has been observed. The binary mixture of ofloxacin and ornidazole is used for treatment of bacterial & parasitic infections. It is used to treat gastrointestinal infections such as acute diarrhea or dysentery, gynecological infections, lung infections and urinary infections.^24^ There are many reported RP-HPLC,^25–27^ HPTLC,^26,28^ capillary zone electrophoresis,^29,30^ voltammetry,^31^ HPLC^32,33^ UPLC tandem mass spectrometry^34^ and spectrophotometry^24,35^ methods for the estimation of ofloxacin and ornidazole from binary pharmaceutical preparations or biological fluids.

In this contribution, we describe the development of easy, cost-effective, fast, green and convenient method ‘to identify the presence of the correct active pharmaceutical ingredients with the stated concentration of each antibiotic drug in the dosage form’ in resource limited areas. Hence, TLC method was employed for separating the antibiotics, visualization of TLC plates was adopted and CCD smartphone camera was employed as detector and finally processing the image with commercially available app software. The proposed method has been applied and compared to standard bench top densitometric method.

## 2. Experimental

### 2.1. Apparatus

- Samsung smartphone S5 CCD camera was used to collect the images.

- Camag Linomat 5 autosampler with Camag micro syringe (100 μL); CAMAG, Muttenz, Switzerland.

- Camag TLC scanner 3 densitometer model 3 S/N 130319 equipped with wincats software for densitometric evaluation; CAMAG, Muttenz, Switzerland.

- Thin-layer chromatographic plates; pre-coated with silica gel 60 F_254_, 20 × 20 cm^2^, 0.25 mm thickness (E. Merck, Darmstadt, Germany).

- UV lamp-short wavelength 254.0 nm, Spectro line®, model CM-10 (Westbury, New York, USA).

### 2.2. Chemicals and reagents

All chemicals and reagent used were of analytical grade. Methanol and *n*-butanol were obtained from Alfa chemical company. NH_4_OH was obtained from PioChem Company. Standards of OFL and ORN were obtained from National Organization for Drug & Control Research. ORNI-O™ tablet was manufactured by India Acme Lite Tech-LLP. Batch number (ALT19317) was purchased from India. Each tablet is claimed to contain 500 mg ORN and 200 mg OFL as active ingredients.

### 2.3. Prepared solutions

#### 2.3.1. Stock standard solutions

A weight equivalent to 10.0 mg of either OFL or ORN were accurately weighted and transferred to 10 mL volumetric flask, dissolved and completed to the mark with the methanol to prepare final stock solution of concentration (1 mg mL^−1^) of the corresponding drugs.

#### 2.3.2. Laboratory prepared mixtures

Different aliquots of OFL and ORN were accurately taken from their standard stock solutions to prepare mixtures containing different ratios of the two drugs.

#### 2.3.3. Chromatographic conditions

##### 2.3.3.1 Mobile phase

Different developing systems were tried, initially, (1) chloroform : ethyl acetate (6 : 4 v/v), (2) chloroform : ethyl acetate : ammonia (7 : 3 : 0.1, by volume), and (3) *n*-butanol : methanol : ammonia (8 : 1 : 0.1, by volume), OFL didn't move from the base line using the previous mentioned systems. By increasing the amount of ammonia to 1.5 mL in the (*n*-butanol : methanol : ammonia) system, OFL moved from the base line. Sharp and symmetric spots were obtained using (*n*-butanol : methanol : ammonia) (8 : 1 : 1.5, by volume) as a developing system where good separation between OFL and ORN with sufficient difference in their (*R*_f_) values without tailing of the separated bands.

##### 2.3.3.2 Visualization of TLC plate

An “iodine chamber” was used for visualization by adding few crystals of solid iodine with powdered silica in a screw-capped TLC chamber. Then, the developed TLC plate (5 × 10 cm^2^) was placed in the iodine chamber for 8 minutes until the spots appeared yellow-brown. After that we used, within 5 minutes, a smartphone camera to detect the intensity of each spot color appeared on the TLC plate using “Color Picker” freely available software application version is 5.0.6 (https://play.google.com/store/apps/details?id=gmikhail.colorpicker) and calculated intensity was utilized for the calibration curve construction for quantitative analysis and also, we made qualitative analysis for the detection of adulterant acetaminophen appearance. It's worth mentioning that iodine is known to be volatile as it sublimes easily, therefore, all the images were collected within 5 minutes once we removed the TLC plates out of the developing jar. The smartphone's rear-facing camera is aligned with a plate guide that places the TLC plate into focus and into the camera field of view. The distance from the camera to the plate is 10 cm.^36^ The TLC plate background was white in color and we capture the image under desk lamp as a source of illumination.

TLC plates also were visualized by certain reagents that are selective for some functional groups, which represent another identification element besides using *R*_f_. To demonstrate that, on a separate plate we employed a selective method for detecting ORN based on visualization by dipping into acidified KMnO_4_ which gives light brown spots after drying, then the “Color Picker” application used to detect the intensity of each spot and calculated intensity was utilized for the calibration curve construction for quantitative analysis.

### 2.4. Pharmaceutical dosage form

In case of TLC-smartphone method, ten tablets were weighed, powdered and the average weight of one tablet was calculated. An accurately weighed powder equivalent to one tablet of ORNI-O™ contains (200 and 500 mg of OFL and ORN, respectively) was transferred to a 100 mL volumetric flask; dissolved in 60 mL methanol as a solvent and sonicated for 15 minutes, the volume was completed with methanol to obtain final concentration of 5000 μg mL^−1^ of ORN and 2000 μg mL^−1^ of OFL. On the other hand, 1 mL was taken from previous flask and was diluted into 10 mL volumetric flask for analyzing ORN to obtain the volume of 500 μg mL^−1^ of ORN. Then 1 mL was taken from the last flask and was diluted into 10 mL volumetric flask for analyzing OFL to obtain the volume of 20 μg mL^−1^ of OFL.

In case of TLC-densitometric method, ten tablets were weighed, powdered and the average weight of one tablet was calculated. An accurately weighed powder equivalent to one tablet had been taken and then 0.01 g of ORNI-O™ tablet was weighted which equivalent to (2000 μg and 5000 μg of OFL and ORN, respectively) and was transferred to 25 mL volumetric flask; dissolved in 10 mL methanol as a solvent and sonicated for 15 minutes, the volume was completed with methanol to obtain final concentration of 80 μg mL^−1^ OFL and 200 μg mL^−1^ ORN. Then 1.25 mL was taken from the previous flask was diluted into 10 mL volumetric flask for analyzing OFL and ORN to obtain the volume of 10 μg mL^−1^ of OFL and 25 μg mL^−1^ of ORN.

## 3. Results and discussion

The TLC visualization technique has many advantages as it is simple, low cost, rapid, available, portable and convenient in resources limited countries, no need for instrumentation or skilled technician, we used conventional TLC plates which is cheaper and we achieved TLC visualization using smartphone for detection and we were able to achieve comparable results by rationale optimization of the developing system, these advantages are on contrary of previous studies which use the HPTLC technique ‘which is expensive and not readily available in the limited resource countries’^28^ and which use smartphones with UV visualization.^36^ It is very convenient to use this technique in the resources limited countries to check if the drug product is adulterated or not without the need of using sophisticated instrumentation, just an image of a plate representing the chromatographic results with the detected spots for visual comparison of *R*_f_ values (identity) and intensities (drug content) to ensure that content in not sub-therapeutic dose.

**Fig. 2:**
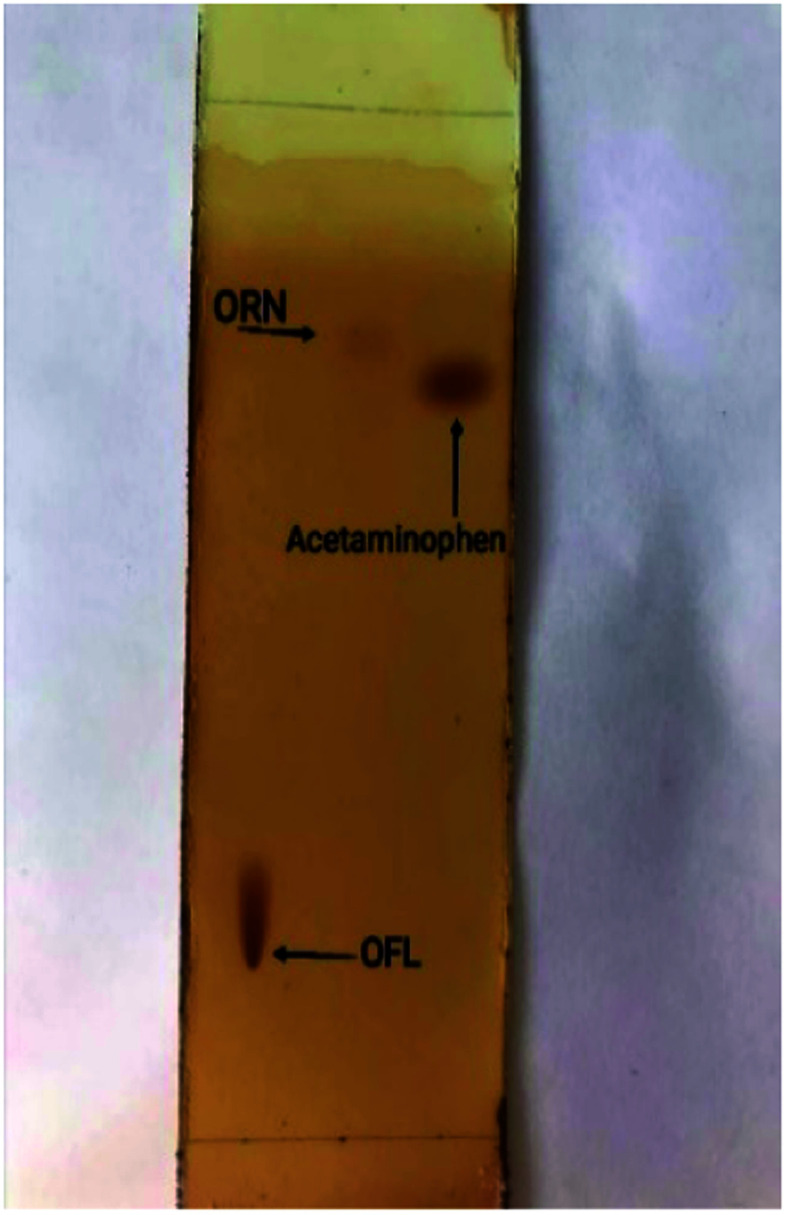
TLC plate showing the three separated compounds; OFL, ORN, acetaminophen visualized with iodine.

TLC plate shows 3 spots of ofloxacin, ornidazole, and acetaminophen (commonly used adulterant) visualized with I_2_ after a run with the mobile phase (*n*-butanol : methanol : ammonia) (8 : 1 : 1.5, by volume) to determine the adulteration of the mixture OFL and ORN with acetaminophen rapidly as shown in Fig. 2. Both OFL and acetaminophen appear as brown spots, while ornidazole appears as a light brown spot. It worth nothing that specific and selective visualization of ORN can be performed with acidified KMnO_4_ reagent which produces yellow changes into light brown color, after drying, which is selective for nitro functional group.

The calculated *R*_f_ for OFL, ORN, acetaminophen is (0.12, 0.76, 0.7), respectively so it's so easy and rapid determining the appearance of adulterant such as acetaminophen (qualitative analysis) only by making visualization with iodine and determine the *R*_f_ of each compound.

To test the capability of the proposed method for quantitative analysis of the antibiotics, we spotted five concentrations of ofloxacin (12.5, 25, 37.5, 50, 62.5 μg/band) on a TLC plate and visualized the separated spots with iodine and on the recorded image we determined the luminance of each spot using the “color picker” freely available software application as shown in Table 1.

**Table 1:** The relation between luminance and different concentrations of OFL and ORN (12.5, 37.5, 50, 62.5 μg/band), (500, 600, 700, 900, 1000 μg/band), respectively

Drug	Concentrations (μg/band)	Average luminance (average of 3 replicates of the same spot)	Standard deviation (SD)	Average luminance (average of three replicates of 3 different spots)	Standard deviation (SD)
OFL	12.5	14.3	0.5	14.4	0.6
	25	12.7	0.5	12.5	0.5
	37.5	10.7	0.5	10.73	0.5
	50	9	0.8	8.9	0.5
	62.5	7	0.6	7.3	0.6
ORN	500	34.3	0.5	34.7	0.6
	600	33.3	0.6	33.4	0.4
	700	31.3	0.5	31.4	0.6
	900	29.3	0.5	28.4	0.5
	1000	26.3	0.5	26.4	0.6

Fig. S1a† represents the relation between calculated luminance and OFL concentration. The relation between concentration of the drug and luminance is linear with *r* = 0.9999 and we spotted five concentrations of ornidazole (500, 600, 700, 900, 1000 μg/band) on a TLC plate and visualized the separated spots with acidified KMnO_4_ and on the recorded image we determined the luminance of each spot using the “color picker” software application as shown in Table 1. Fig. S1b† represents the relation between calculated luminance and ORN concentration. The relation between concentration of the drug and luminance is linear with *r* = 0.9991. This method is sensitive for determination of the antibiotics in their tablet as the concentration in tablet is high (200 mg OFL & 500 mg ORN) but it would be challenging to determine these antibiotics in plasma as the concentration is smaller than TLC-smartphone CCD camera detection limit. Blank was performed on a TLC plates that have been exposed to iodine under the same conditions and the average luminance was 40.0 and the S. D was 0.6.

The validity of the proposed TLC-visualization method was achieved by means of LOD, LOQ, accuracy and precision as shown in Table 2.

**Table 2:** Comparison between validation parameters of the proposed TLC-visualization method and TLC-densitometric method for determination of ofloxacin and ornidazole in their binary mixture

	TLC-visualization method		TLC-densitometric method	
Parameters	OFL	ORN	OFL	ORN
Wave length			320.0 nm	320.0 nm
Range (μg/band)	12.5–62.5	500–1000	5–40	5–50
Linearity (regression equation)	*Y* = −0.1466*x* + 16.183	*Y* = −0.0171*x* + 43.553	*Y* = 434.5*x* + 10 455	*Y* = 516.98*x* + 23 107
Slope^a^	0.15	0.02	434.5	516.9
Intercept^a^	16.2	43.6	10 455	23 107
Correlation coefficient^a^	0.9999	0.9991	0.9999	0.9999
Accuracy^b^ (mean ± SD)	100.0 ± 0.6	100.1 ± 0.7	99.9 ± 0.7	99.7 ± 0.9
Specificity^c^			98.6 ± 0.4	100.5 ± 1.5
LOD (μg/band)^d^	1.6	97.8	0.8	1.1
LOQ (μg/band^d^	4.9	296.3	2.3	3.3
Precision (±RSD%)				
(a) Repeatability^e^	±0.8	±0.7	±0.4	±0.8
(b) Intermediate precision^f^	±0.4	±0.5	±0.3	±0.7

a Average of three determinations.

b Accuracy (the mean of 5 different concentrations of each OFL and OR).

c Recovery of different laboratory prepared mixtures containing different ratios of OFL and OR.

d Limit of detection is determined *via* calculations, LOD = (SD of response/slope) *×* 3.3; LOQ = (SD of response/slope) *×* 10.

e Intraday precision (the RSD of 3 different concentrations) (10, 20, 25 μg/band for OFL) & (5, 10, 50 μg/band for OR) in (TLC-densitometric) and (12.5, 25, 37.5 μg/band for OFL) & (500, 700, 900 μg/band for OR) in TLC-smartphone, 3 replicates each, within the same day.

f Interday precision (the RSD of 3 different concentrations) (10, 20, 25 μg/band for OFL) & (5, 10, 50 μg/band for OR) in (TLC-densitometric) and (12.5, 25, 37.5 μg/band for OFL) & (500, 700, 900 μg/band for OR) in TLC-smartphone, 3 replicates each, on 3 successive days.

The TLC-smartphone method was successfully applied for the determination of OFL and ORN in their combined pharmaceutical formulation (ORNI-O™ tablet) and the recovery results was 99.1 ± 0.7 for ofloxacin and 98.4 ± 0.6 for ornidazole as shown in Table 3.

**Table 3:** Determination of OFL and ORN in ORNI-O™ tablet by TLC-smartphone method and the TLC-densitometric

Pharmaceutical formulation	aRecovery% ± SD (TLC-smartphone method)		aRecovery% ± SD (TLC-densitometric method)	
bORNI-O™	OFL	ORN	OFL	ORN
	99.1 ± 0.7	98.4 ± 0.6	98.3 ± 0.5	99.9 ± 0.6

a Average of three determinations.

b Batch no. ALT19317 (labeled to contain 200 mg OFL and 500 mg ORN).

**Fig. 3:**
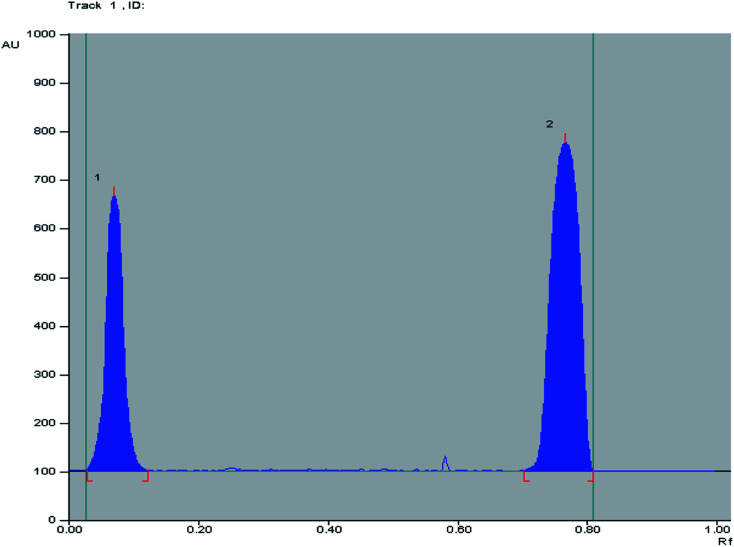
2D TLC chromatogram of (1) ofloxacin (*R*_f_ = 0.12) and (2) ornidazole (*R*_f_ = 0.76), at 320.0 nm using *n*-butanol : methanol : ammonia (8 : 1 : 1.5, by volume) as a developing system.

The proposed method has been compared to the bench top TLC-densitometry method which is a useful technique for the resolution and in turn for the quantitative determination of drug mixtures. This method offers high sensitivity and selectivity for the analysis of OFL and ORN in the presence of each other without any interference in their pure form and in their pharmaceutical dosage form. Different developing systems were tried, chloroform : ethyl acetate (6 : 4 V/V), chloroform : ethyl acetate : ammonia (7 : 3 : 0.1, by volume), and *n*-butanol : methanol : ammonia (8 : 1 : 0.1, by volume), OFL didn't move from the base line using the previous mentioned systems. Increasing the amount of ammonia to 1.5 mL made the OFL moves from the base line. Sharp and symmetric spots were obtained using (*n*-butanol : methanol : ammonia) (8 : 1 : 1.5, by volume) as a developing system where good separation between OFL and ORN with sufficient difference in their (*R*_f_) values without tailing of the separated bands as shown in Fig. 3 was obtained. Well defined bands were obtained when the chromatographic tank was previously saturated with the mobile phase for 20 minutes at room temperature. The instrumental conditions such as slit dimension and detection wavelengths were optimized. Detection at *λ* 320 nm for both OFL and OR was suitable providing good sensitivity for determination of OFL and OR with minimal noise. TLC-densitometric scanning chromatograms of different concentrations of OFL and ORN were performed. Calibration curves were constructed representing the relationship between the obtained integrated peak areas and the corresponding concentrations in the range of (5–40 μg/band) for OFL and (5–50 μg/band) for OR. Linear relationships were obtained as shown in Fig. S2a and b.† The regression equations were computed and found to be:PA_OFL 320.0 nm_ = 434.5*x* + 10455 *r* = 0.9999PA_OR 320.0 nm_ = *Y* = 516.98*x* + 23107 *r* = 0.9999where PA is the integrated peak area at 320.0 nm for OFL and OR, *x* is the corresponding concentration in μg/band and *r* is the correlation coefficient.

The validity of the TLC densitometric method was achieved by means of LOD, LOQ, accuracy and precision as shown in Table 2. The results obtained from the laboratory prepared mixtures containing different ratios of OFL and ORN separated at specified conditions were presented in Table 4. The TLC-densitometric method was successfully applied for the determination of OFL and ORN in their combined pharmaceutical formulation (ORNI-O™ tablet) and the recovery results was 98.3 ± 0.5 for ofloxacin and 99.9 ± 0.6 for ornidazole as shown in Table 3.

**Table 4:** Determination of ofloxacin and ornidazole in their laboratory prepared mixtures by the TLC-densitometric method

Mix. ratio	aRecovery%	
OFL : OR	OFL	ORN
10 : 10	98.1	100.8
10 : 20	98.5	98
20 : 10	98.5	100.6
10 : 25	98.6	102.3
40 : 10	99.3	100.7
Mean	98.6	100.5
SD	0.4	1.5

a Average of three determinations.

Statistical comparison of the results obtained by the proposed method and those by applying the “official” and “reported” methods showed that there is no significant difference with respect to accuracy and precision as represented in Table 5. Where the proposed method is the TLC-smartphone method. In case of OFL there is an “official” method (Pharmacopeial method), therefore it has been adopted for comparison, whereas, in case of ORN there is no official method so the “reported” densitometric method had been used to compare the ORN results with TLC-smartphone method.

**Table 5:** Statistical analysis of the results obtained by the proposed method and the official method for the determination of OFL and ORN in pure powder form

Item	OFL		ORN	
	Proposed method^a^	Official method^b^	Proposed method	Reported method^c^
Mean	100.1	100.4	100.7	99.7
SD	0.9	0.8	0.8	0.9
Variance	0.9	0.7	0.6	0.8
*N*	5	6	5	5
Student's *t*-test^d^	0.39 (2.3)		0.72 (2.36)	
*F*-value^d^	1.2 (5.41)		1.34 (9.12)	

a Proposed method is the TLC-smartphone method.

b Potentiometric method by dissolving 0.3 g of OFL in 100 mL of anhydrous acetic acid. Titrate with 0.1 M perchloric acid and determine the end point.^37^ (pharmacopeial method which readings of OFL were compared with).

c Reported method is the method which ORN readings were compared with, as ORN has no official method.^38^

d Numbers between parentheses represents the corresponding tabulated values of *t* and *F* at *P* = 0.05.

## 4. Conclusion

Low quality medication in low-income countries represent a major challenge for healthcare system, therefore, developing simple, robust method for detecting low quality and counterfeit medications will have a major impact on population health. TLC separation method combined with simple I_2_ visualization method and smartphone detection for capturing and analysis of the image has been proposed for both qualitative and quantitative determination of antibiotics drugs in bulk and pharmaceutical formulation. Furthermore, based on the specific functional group such as nitro, ornidazole can be independently identified in presence of other co-formulated drug and possible adulterant such as “acetaminophen”. The method was compared to the benchmark densitometric method and no statistical difference was reported. This TLC separation plus smartphone detection method is very simple, cost-effective (no UV source is required) and more available so it can be extended to detect other antibiotics in pharmaceutical formulations.

## Conflicts of interest

There are no conflicts of interest to declare.

## Supplementary Material

RA-011-D1RA01346G-s001
