# Supplementary material for: TLC-smartphone in antibiotics determination and low-quality pharmaceuticals detection
Source: RSC Adv. 2021 May 26;11(31):19196–202. doi: 10.1039/d1ra01346g (PMC9033552; doi:10.1039/d1ra01346g)
Supplement: RA-011-D1RA01346G-s001 [file RA-011-D1RA01346G-s001.pdf]

Electronic Supplementary Material (ESI) for RSC Advances.
This journal is © The Royal Society of Chemistry 2021

Electronic Supplementary Information

for

"TLC - Smartphone in Antibiotics Determination and Low-Quality Pharmaceuticals Detection"

Asmaa G. Gad[a], Yasmin Mohammed Fayez[b], Khadiga M. Kelani[a,b], Amr M. Mahmoud[b*]

[a]Analytical Chemistry Department, Faculty of Pharmacy, Modern University for Technology and Information, Cairo, Egypt

[b]Analytical Chemistry Department, Faculty of Pharmacy, Cairo University, El-Kasr El-Aini Street, 11562 Cairo, Egypt

*Corresponding author email: amr.bekhet@pharma.cu.edu.eg

Figure S1:

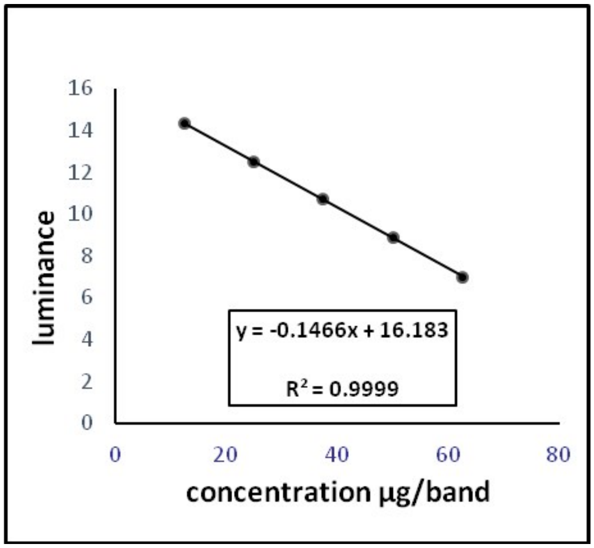

(S1a)

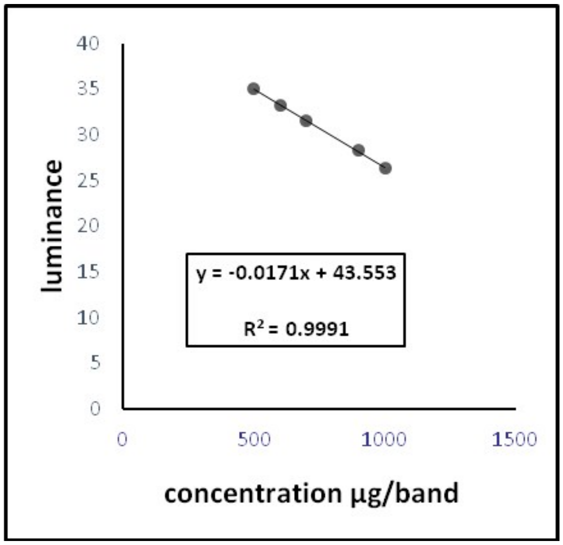

(S1b)

Figure S1. (S1a) The relation between OFL concentration per spot and the calculated luminescence of recorded image using CCD smartphone camera. (S1b) The relation between ORN concentration per spot and the calculated luminescence of recorded image using CCD smartphone camera. The points on the graphs are the mean value of three replicates.

Figure S2:

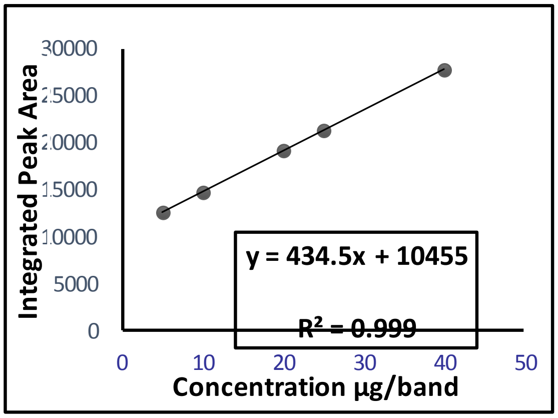

(S2a)

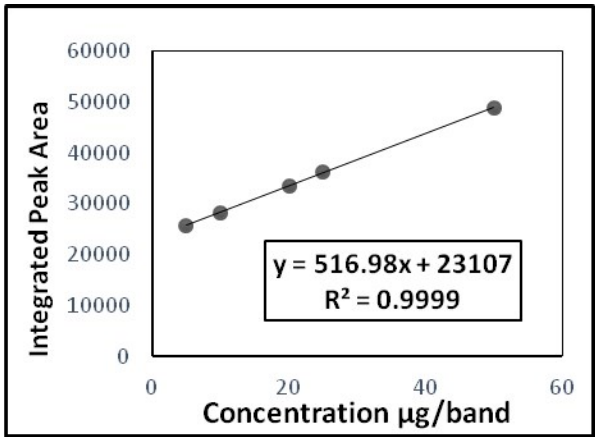

(S2b)

Figure S2. (S2a) Linearity of the peak area to the corresponding concentrations of Ofloxacin in concentration range (5-40 µg/band) at 320.0 nm using n-butanol: methanol: ammonia (8:1:1.5, by volume) as a developing system. (S2b) Linearity of the peak area to the corresponding concentrations of Ornidazole in concentration range (5-50 µg/band) at 320.0 nm using n-butanol: methanol: ammonia (8:1:1.5, by volume) as a developing system.
